# Preoperative cognitive impairment predicts deep anaesthesia and higher postoperative pain in elderly patients: an observational study

**DOI:** 10.1186/s12871-025-03452-w

**Published:** 2025-10-31

**Authors:** Julian Runge, Rosa K. Heedfeld, Carla D. Grundmann, Jan M. Wischermann, Petra Bischoff, Ulrich H. Frey

**Affiliations:** 1 Department of Anaesthesiology, Operative Intensive Care Medicine, Pain and Palliative Medicine, Marien Hospital Herne, Ruhr-University Bochum, Hölkeskampring 40, Herne, Germany; 2 Clinic for General and Visceral Surgery, HOCH Health Ostschweiz, Spital Wil, Wil, CH-9500 Switzerland

**Keywords:** Preoperative cognitive impairment, Anaesthetic sensitivity, Quantitative electroencephalogram analysis, Postoperative pain, Delirium

## Abstract

**Background:**

The prevalence of cognitive impairment and its associated risks under general anaesthesia increases with age. This necessitates careful observation and mitigation of anaesthetic risk in elderly patients. While deeper anaesthetic states are hypothesised to predispose patients to postoperative delirium, the causal mechanisms remain unclear, particularly concerning age-related sensitivity to anaesthetics and cognitive deficits.

**Methods:**

This study aimed to investigate the relationship between preoperative cognitive impairment and variability in anaesthetic sensitivity. Additionally, it evaluates the potential of electroencephalogram parameters as markers for identifying cognitive impairment. 84 patients aged ≥ 65 years who underwent elective urological surgery lasting ≥ 60 min were included in this exploratory observational study. Preoperative cognitive function was assessed using the Montreal Cognitive Assessment (MoCA). Cardiovascular and respiratory parameters, anaesthetic concentrations, electroencephalogram indices, and burst suppression ratios were recorded digitally. The primary outcome was the correlation between MoCA scores and deep anaesthesia levels. Secondary outcomes included quantitative electroencephalogram analysis, postoperative pain, and incidence of delirium.

**Results:**

Of the 84 patients screened, 67 were included (median age: 70 [67 to 74] years; median total procedure time: 200 [166 to 247] minutes). The mean MoCA score was 24.3 (± 3.2) points. Cognitive impairment (MoCA score < 26) was identified in 64.2% of the patients. Lower MoCA scores correlated with prolonged periods of deep anaesthesia (ρ=-0.27; *P* = 0.027). Severe cognitive impairment was associated with longer durations of deep anaesthesia (20.1% vs. 1.1% of the monitoring window; *P* = 0.006). Burst suppression occurred in 31.3% of the patients but showed no association with MoCA scores. Cognitive impairment was linked to increased postoperative pain requiring treatment (*P* = 0.001), but not to delirium incidence.

**Conclusions:**

Severe preoperative cognitive impairment was associated with prolonged episodes of deep anaesthesia despite comparable anaesthetic dosages, suggesting heightened sensitivity in these patients. Further studies are needed to determine whether these findings can serve as markers for identifying cognitive impairment in preoperative assessments.

**Supplementary Information:**

The online version contains supplementary material available at 10.1186/s12871-025-03452-w.

## Introduction

The incidence of cognitive impairment (CI) increases significantly with age, affecting approximately 24% of individuals aged ≥ 65 years [1]. This demographic trend underscores the importance of identifying and mitigating the risks associated with general anaesthesia in the elderly population during clinical practice. In this context, intraoperative electroencephalogram (EEG) monitoring has become a fundamental aspect of anaesthetic care, particularly for high-risk patients, providing multiple benefits to clinical outcomes [2–4].

However, despite its growing use, specific recommendations for routine use remain limited to high-risk groups based on the current evidence.

Physiological changes associated with aging contribute to heightened sensitivity to anaesthetics [5], which manifests as altered EEG patterns during surgery [6]. Nonetheless, definitive determination of the extent to which age-related heightened sensitivity to anaesthetics, in the form of an altered dose-response relationship, contributes to this phenomenon remains elusive.

Emerging evidence suggests that certain anaesthetics such as propofol and sevoflurane inhibit thalamocortical circuits while inducing alpha-band oscillations (8–12 Hz) in the prefrontal cortex [7–10]. These EEG changes have been associated with postoperative cognitive decline and delirium risk. However, the clinical implications of these findings, particularly their relevance to patients with pre-existing CI, have not been fully elucidated. It has been hypothesised that deeper stages of anaesthesia may increase vulnerability to postoperative complications such as delirium. Delirium is a well-documented predictor of increased morbidity, mortality, and long-term cognitive decline in elderly patients undergoing surgery [11–14].

Despite its clinical significance, showing that preoperative CI is a risk factor for postoperative delirium [1, 15], CI is often excluded from preoperative risk assessment models designed to predict postoperative outcomes [16–19].

CI has been associated with alpha band activity (alpha reduction), based on EEG findings [20–22]. The extent to which cognitive deficits are also associated with an increased occurrence of burst suppression (BS) has not been adequately investigated.

To address these gaps, the present study investigated whether preoperative CI, as measured using the Montreal Cognitive Assessment (MoCA), correlates with increased anaesthetic sensitivity and distinctive EEG signatures during surgery. We further explored the potential of EEG parameters as reliable biomarkers for identifying functional CI, thereby enhancing perioperative risk stratification.

## Materials and methods

### Study design and population

This exploratory observational study included 84 patients aged ≥ 65 years who underwent elective urological surgeries lasting ≥ 60 min from June 2020 to November 2020. The study protocol was approved by the Ethics Committee of the Medical Faculty at Ruhr University Bochum (approval number: 19–6795, date of approval: 19/03/2020). The patients provided written informed consent to participate in the study. Data were extracted using the Philips IntelliSpace and Critical Care and Anaesthesia reporting system (ICCA, Philips Medical Systems, Andover, MA, USA).

### Inclusion and exclusion criteria

Patients were eligible for inclusion if they were aged ≥ 65 years and scheduled for elective urological surgery lasting ≥ 60 min, conducted under balanced anaesthesia with sevoflurane. Exclusion criteria included emergency interventions, pre-existing neurological conditions with structural brain defects, substance abuse, language barriers, or refusal to participate in the study.

### Preoperative cognitive assessment

Preoperative cognitive function was evaluated using the MoCA score, a validated tool for patients aged 55–85 years old. The MoCA scores ranged from 0 to 30 points, with scores ≥ 26 indicating normal cognitive function [23]. In accordance with prior literature, we defined cognitive impairment as MoCA score < 26 points and severe impairment as MoCA score ≤ 21 points. These thresholds were specified a priori based on established validation studies [24, 25].

### Anaesthetic management

Anaesthesia was administered according to the standard hospital protocols. Preoperative laboratory tests were performed, including those for serum creatinine and hemoglobin concentrations. According to hospital standards, pharmacological premedication was administered only in exceptional cases. General anaesthesia was induced intravenously with fentanyl (2–4 µg kg^−1^) and propofol (1.5–2.5 mg kg^−1^), followed by rocuronium (0.5–0.6 mg kg^−1^) for muscle relaxation and endotracheal intubation. Maintenance of anaesthesia involved a balanced administration of sevoflurane. Intraoperative opioid supplementation was achieved using additional fentanyl boluses (25–50 µg) as required to maintain haemodynamic stability and surgical tolerance. Neuromuscular blockade maintained as clinically indicated; Neuromuscular function was continuously monitored using train-of-four (TOF) stimulation throughout the procedure, and full recovery was confirmed before extubation. Epidural anaesthesia was employed selectively based on surgical risk-benefit assessments using catheter technique. In accordance with hospital standards, particular attention was paid to hemodynamic stability (normotension, defined as a mean arterial pressure > 65 mmHg or higher in cases of cardiac comorbidity) during the induction and maintenance of anaesthesia. Intraoperatively, body temperature was monitored in all patients. The Surgical Apgar Score (SAS) was recorded for each patient to estimate possible imminent postoperative complications [26–28].

### EEG monitoring

Intraoperative electroencephalogram (EEG) monitoring was performed using a Narcotrend monitor (Narcotrend-Gruppe, Hannover, Germany). The Narcotrend index (NI) ranged from 100 (wakefulness) to 0 (deep anaesthesia), and phases of BS were calculated to quantify suppressed EEG signals (< 5 or < 10 µV amplitudes lasting > 240 µs within the preceding 60 s). The Narcotrend system recorded EEG data at a sampling rate of 256 Hz using built-in hardware filters (band-pass 0.5–45 Hz). For electrode placement, the two measurement electrodes were positioned with a minimum distance of 8 cm, and an additional reference electrode was applied. Electrode impedance was checked internally by the device before each recording; if impedance values ≥ 6 kΩ were detected, the respective electrode was reapplied. Artefact rejection was performed automatically by the device; excluding epochs with excessive noise or electrode disturbance. epochs with movement or electrode noise exceeding predefined amplitude thresholds were excluded. Recordings with more than 10% artefactual or missing signal time were omitted from analysis. In addition, the timestamps recorded by the Narcotrend monitor and the digital anaesthesia information system were carefully checked for consistency prior to induction of anaesthesia and subsequently synchronized using these time references to ensure accurate temporal alignment of all collected data.

### Anaesthetic depth assessment

An end-expiratory sevoflurane concentration corresponding to 1.0 age-adjusted minimum alveolar concentration (aaMAC_et_; age-adjusted end-expiratory minimum alveolar concentration of sevoflurane, calculated according to the Mapleson formula) was initially targeted to achieve an orientative EEG-based anaesthesia depth with NI values of approximately 60–40 [29]. In cases where NI values dropped below 40 despite stable aaMAC_et_ and haemodynamic stability, anaesthetic doses were adjusted cautiously to maintain balance between adequate anaesthetic depth, haemodynamic stability, and surgical requirements. Values for aaMAC_et_ were recorded to determine the anaesthetic dose-response relationships. Controlled ventilation ensured normoventilation with an end-expiratory carbon dioxide partial pressure target of 36 mmHg.

### Postoperative assessments

Postoperative evaluations began in the recovery room immediately after surgery (T0) and continued twice daily on postoperative days 1–4 (T1–T4). Responsible trained nursing staff assessed the patients using the German version of the Confusion Assessment Method for Intensive Care Units (CAM-ICU) [30]. The agitation or sedation status was assessed using the Richmond Agitation Sedation Scale (RASS) [31]. Nausea, delirium screening using the CAM-ICU criteria, and sedation levels using the RASS were assessed. Pain levels at rest and during movement were assessed using a visual analogue scale (0–100). For patients with cognitive impairment, trained nursing staff provided repeated prompts and clarifications.

### EEG analysis window selection

EEG analysis focused on a time window extending from 15 min after surgical incision to 30 min before suturing. The minimum time interval between the induction dose of propofol (eliminating the induction effects of propofol, i.e., EEG suppression) [30, 32] and the administration of surgical stimulation was 30 min. However, a 30-minute interval between the administration of propofol and the potential reduction of anaesthetics before anaesthesia withdrawal is also recommended. This analysis window was chosen to ensure stable anaesthetic conditions and to minimize artefacts, thereby avoiding suppression effects from the induction dose of propofol, immediate artefacts from surgical stimulation, and potential anaesthetic reduction prior to emergence; sensitivity differences outside this window were not assessed.

### Study outcomes

The primary outcome was the correlation between the MoCA scores and deep anaesthetic states, defined by NI values < 40. The secondary outcomes included the frequency of BS, postoperative pain requiring treatment, nausea and vomiting (PONV), and delirium incidence.

### Statistical analysis

Data were tested for normal distribution using the Shapiro-Wilk test. Descriptive statistics included medians and quartiles for non-normally distributed data and mean ± standard deviation for normally distributed data. Correlations were analysed using Spearman’s rank correlation coefficient for non-parametric data and Eta coefficients for metric-nominal relationships. Group comparisons were performed using the t-test for normally distributed variables, Mann-Whitney U test for non-normally distributed data, and chi-square test for nominal variables. Binary logistic regression assessed MoCA scores as predictors of deep anaesthesia (NI < 40). All data points were included; no outliers were excluded. Analyses were performed on complete cases. Missing data were not imputed. Patients with missing EEG data or incomplete intraoperative documentation were excluded prior to analysis, as detailed in the Results section. Statistical significance was set at *P* ≤ 0.05, with two-sided testing applied. All analyses were conducted using SPSS 28.0 for windows (IBM, Armonk, NY, USA).

## Results

Of the 84 patients screened, 67 were included in the final analysis. The reasons for exclusion included the use of desflurane as the anaesthetic agent (*n* = 2), missing EEG data (*n* = 10), artifact-rich EEG recordings (*n* = 2), and incomplete intraoperative documentation (*n* = 3).

The median age of the study cohort was 70 years [67 to 74], with 94% male participants (*n* = 63). The median body mass index (BMI) was 26.3 kg/m^2^ [24.5 to 28.7], which corresponds to the overweight category. The patients underwent elective urological surgeries including robot-assisted radical prostatectomy (*n* = 50; 74.6%), radical cystectomy (*n* = 10; 14.9%), and other procedures (*n* = 7; 10.4%). Balanced general anaesthesia with sevoflurane was administered to all patients, and epidural anaesthesia (EDA) was used in 20.9% (*n* = 14) of the cases. The median duration of surgery was 200 [166 to 247] minutes and the median SAS was 8 [7 to 9]. The EEG analysis window had a median duration of 151 min [124 to 203], and the average mean arterial pressure (MAP) during this period was 87.4 (± 8.1) mmHg. The additional demographic and intraoperative variables are summarised in Table 1.

**Table 1:** Demographical and intraoperative variables

Variable	Value
	*n* = 67
Age in years	70 [67 to 74]
Gender male	63 (94)
Body mass index in kg·m^−2^	26.3 [24.5 to 28.7]
Type of surgery	
- Robot-assisted radical prostatectomy	50 (74.6)
- Radical cystectomy	10 (14.9)
- other	7 (10.4)
SAS	8 [7 to 9]
Use of EDA (yes)	14 (20.9)
Preoperative Hb in g·dl^−1^	14.5 [13.8 to 15.4]
Preoperative creatinine value in mg·dl^−1^	1 [0.9 to 1.1]
Duration of surgery (incision-suture) in minutes	200 [166 to 247]
Duration of EEG analysis window in minutes	151 [124 to 203]
Mean arterial pressure in mmHg	87.4 ± 8.1
Intraoperative volume administered in ml	2000 [1400 to 2100]
Blood loss in ml	200 [100 to 475]
Intraoperative minimal temperature in °C	36.0 [35.6 to 36.3]
Intraoperative maximal temperature in °C	36.9 [36.5 to 37.2]
Occurrence of intraoperative hypotension < 65 mmHg	16 (23.9)

Data are mean ± SD, median [IQR], and n (%)

*SAS* Surgical Apgar Score, *EDA* Epidural anaesthesia

**Fig. 1:**
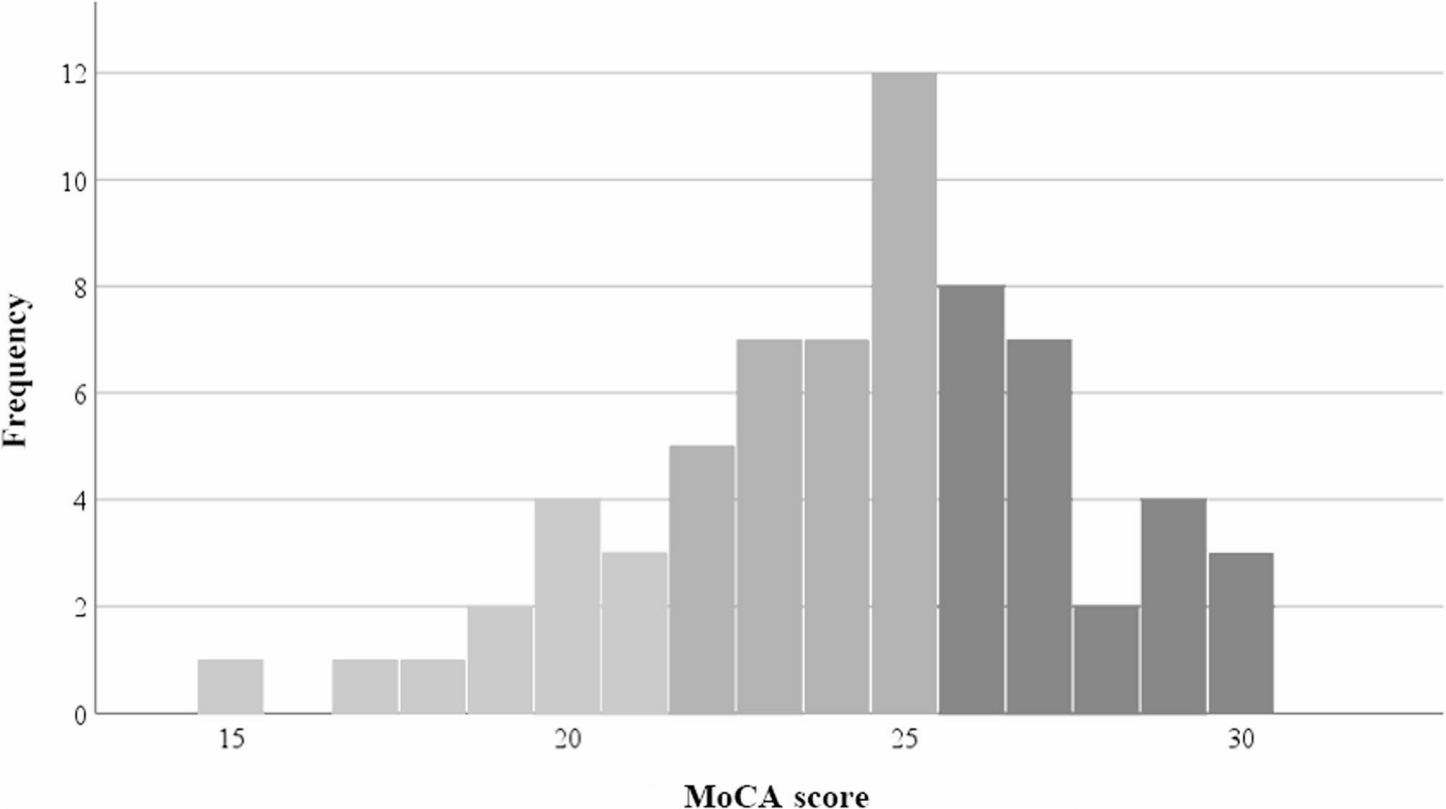
Frequency distribution of MoCA scores Legend:* n* = 76. Maximum MoCA score 30 points. CI: MoCA score < 26, severe CI: MoCA score ≤ 21. Mean MoCA score 24.3 ± 3.2 MoCA = Montreal Cognitive Assessment; CI = cognitive impairment

The mean MoCA score for the study cohort was 24.3 (± 3.2) points. Cognitive impairment, defined as a MoCA score of < 26, was present in 64.2% of the patients (*n* = 43). Severe cognitive impairment (MoCA score ≤ 21) was observed in 17.9% of patients (*n* = 12). The frequency distribution of MoCA scores is shown in Fig. 1.

### EEG parameters and dose-response relationships

Descriptive analyses of intraoperative EEG parameters and dose-response relationships during the analysis window are presented in Table 2.

**Table 2:** Analysis parameters for EEG (NI; BS) and dose-response relationship

Variable	Value
	*n* = 67
aaMAC_et_	1.1 [1.0 to 1.3]
NI	47.2 ± 9.9
NI/aaMAC_et_	41.1 ± 9.2
NI < 40 in %	3.3 [0 to 20.6]
NI 60 − 40 in %	74.8 [54.5 to 90.9]
NI > 60 in %	0.1 [0 to 9.2]
NI < 40 in minutes	5.3 [0 to 33.3]
NI 60 − 40 in minutes	111.3 [65 to 146]
NI > 60 in minutes	0.3 [0 to 6]
Sevoflurane_et_ in Vol%	2 [1.8 to 2.3]
Occurrence of BS	21 (31.1)

Data are mean ± SD, median [IQR], and n (%)

*aaMAC*_et_ Age-adjusted end-expiratory minimum alveolar concentration of sevoflurane, calculated according to the Mapleson formula, *NI* Narcotrend index, *BS* Burst suppression

The median aaMAC_et_ was 1.1 [1.0 to 1.3]. The mean NI in the study cohort was 47.2 ± 9.9, and the dose-response relationship shown by NI/aaMAC_et_ was 41.1 ± 9.2. Deep anaesthesia states with NI < 40 occurred for a median duration of 5.3 min [0 to 33.3], corresponding to a median percentage of monitoring time of 3.3% [0 to 20.6]. The phases of BS were observed in 21 patients (31.3%).

### Primary outcome: correlation between MoCA scores and anaesthetic depth

**Fig. 2:**
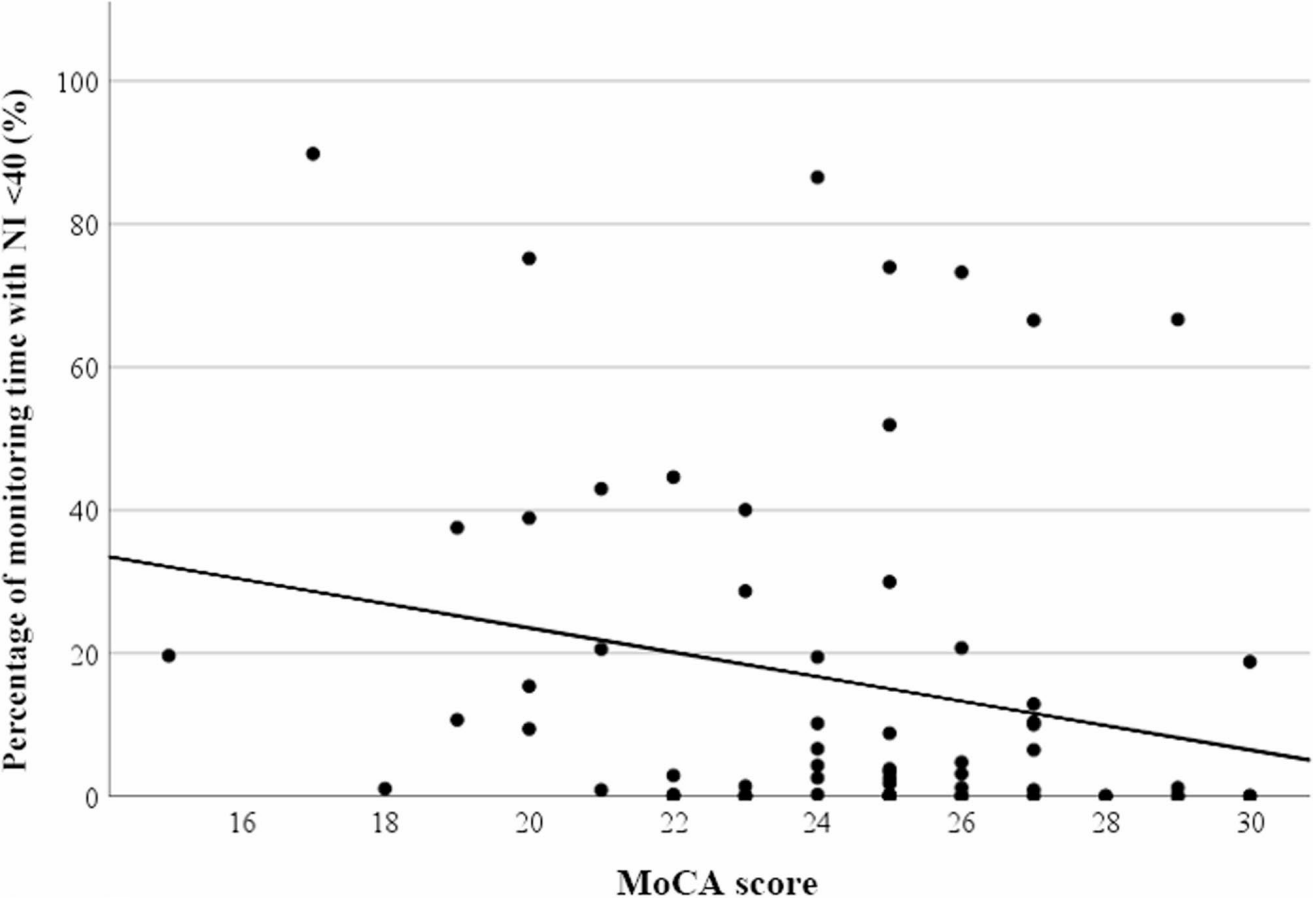
Correlation between deep anaesthesia stages (NI < 40; % of monitoring time) and the MoCA score Legend: Patients with low MoCA scores showed a significantly higher percentage of deep anaesthesia stages in the time window total. *n* = 67; ρ=−0.270; *P* = 0.027 MoCA = Montreal Cognitive Assessment; NI = Narcotrend index

A significant negative correlation was identified between the MoCA scores and the percentage of time spent in deep anaesthesia states (NI < 40) during the analysis window (ρ=−0.270; *P* = 0.027; Fig. 2). Similarly, a significant negative correlation was found between MoCA scores and the absolute duration of deep anaesthesia in minutes (ρ=−0.247; *P* = 0.044).

Binary logistic regression analysis demonstrated that lower MoCA scores were significantly associated with an increased likelihood of deep anaesthesia states with an NI < 40 [OR 1.22; 95%-CI 1.01 to 1.47; per point increase in preoperative MoCA score for the occurrence of deep anaesthesia (NI < 40); *P* = 0.039] (Table 3).

**Table 3:** Primary endpoint. Binary logistic regression: association between preoperative MoCA scores (continuous predictor) and the occurrence of deep anaesthesia (NI < 40, yes/no). OR per point increase in preoperative MoCA score for the occurrence of deep anaesthesia (NI < 40)

	Dependent variable				
	Occurrence of deep anaesthesia (NI < 40, yes/no)				
Independent variable	*n*	OR	95%-CI	*P*	Overall percentage correctly classified
Preoperative MoCA score (continuous)	67	1,22	1,01–1,47	0,039	68,7%

*NI* Narcotrend index, *OR* Odds ratio, *95%-CI* 95% confidence interval, *MoCA* Montreal Cognitive Assessment

No significant correlations were found between the MoCA scores and demographic or intraoperative variables, including age, duration of surgery, SAS points, type of surgery, occurrence of intraoperative hypotension, use of epidural anaesthesia, or preoperative haemoglobin or creatinine levels (Supplement Table A).

Furthermore, correlation analyses revealed no substantial associations between the occurrence of intraoperative deep anaesthesia (NI < 40) and demographic or intraoperative variables. Eta-square coefficients and corresponding p-values consistently indicated very weak relationships. Notably, age, body mass index, SAS score, preoperative creatinine, mean arterial pressure, and occurrence of intraoperative hypotension demonstrated negligible effect sizes and non-significant results.

Although intraoperative administered volume (η²=0.069; *P* = 0.032), blood loss (η²=0.104; *P* = 0.009), intraoperative minimal temperature (η²=0.077; *P* = 0.025), and maximal temperature (η²=0.008; *P* = 0.048) reached statistical significance, the corresponding effect sizes were consistently small, indicating only limited explanatory value (Supplement Table B).

**Fig. 3:**
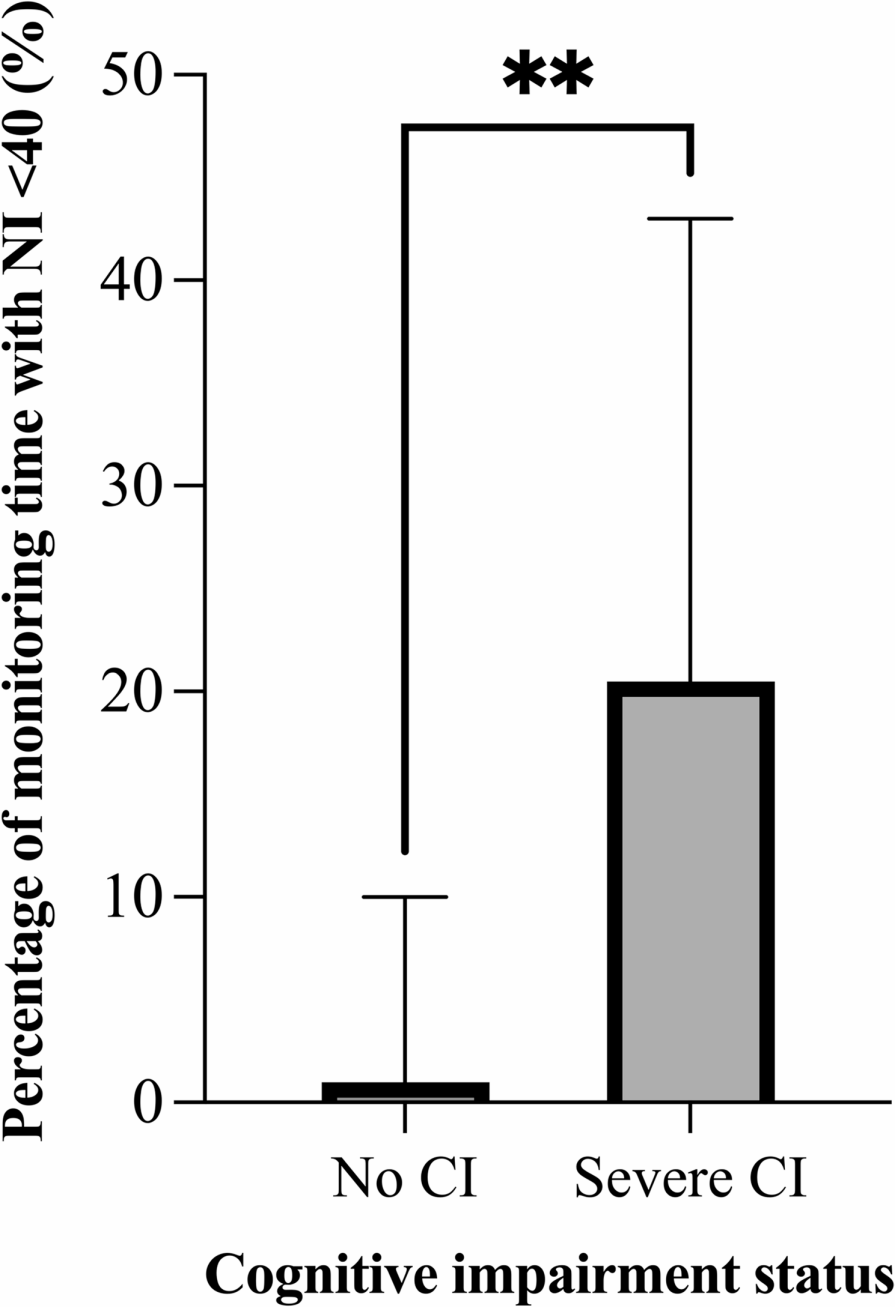
Cognitive impairment status and duration in deep anaesthesia Legend: Patients without CI (MoCA score ≥ 26, *n* = 24) spent significant lower percentage of monitoring time in deep anaesthesia (NI < 40) compared to patients with severe CI (MoCA score ≤ 21, *n* = 12), 1.1% [IQR 0 to 12.9] vs. 20.1% [IQR 9.7 to 41.9], *P* = 0.006 MoCA = Montreal Cognitive Assessment; CI = cognitive impairment; NI = narcotrend index

Patients with severe cognitive impairment (MoCA score ≤ 21, *n* = 12) exhibited significantly longer durations in deep anaesthesia states compared to those without cognitive impairment (*n* = 24), both as a percentage of monitoring time (20.1% vs. 1.1%, *P* = 0.006, Fig. 3) and absolute duration in minutes (21.0 vs. 2.3 min; *P* = 0.012). However, no differences were observed in the aaMAC_et_ values between the groups.

### Secondary outcomes

Postoperative nausea and vomiting (PONV) occurred in 31.3% of patients (*n* = 21) during postoperative days T0 to T4. Pain at rest requiring treatment (VAS score > 30) occurred at a median frequency of 13.5% on the day of surgery (T0) and increased to a median frequency of 15.8% during days T1–T4.

Acute psychological changes (RASS) were detected in 26.9% of patients (*n* = 18), while delirium assessed using the CAM-ICU criteria was documented in only four patients (6%) on postoperative day T0.

A significant correlation was observed between decreasing MoCA scores and increased occurrence of pain at rest requiring treatment on postoperative day T1 (η²=0.064; *P* = 0.041). A highly significant correlation (η²=0.198; *P* = 0.001) was also found between decreasing MoCA scores and pain at rest that required treatment on postoperative day T3.

Patients with severe cognitive impairment exhibited significantly higher frequencies of pain requiring treatment on postoperative day T1 than those without cognitive impairment. No significant correlations were found between MoCA scores and the occurrence of PONV or delirium (Table 4).

**Table 4:** Secondary endpoints. Correlations between the MoCA scores and the data of the postoperative visits (T0-T4)

Variable	η²	*P*
Nausea (T0-T4)	0.024	0.210
Delirium (T0-T4)	0.001	0.842
Pain at rest (requiring treatment)		
- T0	0.001	0.820
- T1	0.064	0.041
- T2	0.002	0.724
- T3	0.198	0.001
- T4	0.045	0.172

*PONV* Post-operative nausea and vomiting, *T0* recovery room, *T1-T4* Visit on postoperative days 1–4, *η*² Eta-squared coefficient

## Discussion

In this study, we showed that preoperative cognitive impairment (CI), as assessed by the MoCA scores, is significantly associated with prolonged durations of deep anaesthesia during elective urological surgeries. Patients with severe CI (MoCA score ≤ 21) exhibited markedly longer periods of deep anaesthesia (NI < 40) than those without CI, despite receiving comparable doses of anaesthetic agents. Further, also binary logistic regression analysis demonstrated that lower MoCA scores were significantly associated with an increased likelihood of deep anaesthesia states with an NI < 40. These findings suggest that patients with CI may exhibit heightened sensitivity to anaesthetics, which could have implications for perioperative management.

Interestingly, while BS was observed in 31.3% of the patients, no significant association was found between the frequency of BS and MoCA scores. Furthermore, although CI was associated with increased postoperative pain requiring treatment, no significant correlation was identified between CI and the incidence of postoperative delirium or nausea/vomiting.

In the context of anaesthetic procedures targeting the brain, the monitoring of cognitive functions is often neglected in favour of acute vital bodily functions.

This oversight is notable given the documented evidence from prior studies highlighting age-related CI as a significant predictor of postoperative complications, particularly delirium development [1, 15, 33]. Increased sensitivity to anaesthetics, which is reflected in the EEG by a tendency toward comparatively deeper anaesthetic stages and a higher incidence of BS, is also associated with a deterioration in outcome in studies [12, 13, 34, 35].

Little is known about the relationship between CI and increased sensitivity to anaesthetics. A comprehensive understanding of cognitive impairments is mandatory for effectively managing the expanding demographics of elderly patients in clinical anaesthesiology. This is particularly relevant for adjustment of individual preoperative risk profiles.

However, the MoCA test, which has proven to be a sensitive test procedure for detecting CIs, particularly in geriatric research [24], can only be carried out at a great expense in terms of time and personnel.

### Dose-response relationship

The observed association between CI and prolonged deep anaesthesia is in line with previous studies suggesting that age-related changes in brain physiology increase the sensitivity to anaesthetic agents. EEG patterns, such as reduced alpha-band oscillations and increased BS, have been linked to postoperative cognitive decline; however, their relationship with the preoperative cognitive status remains poorly understood [20–22]. Our findings highlight the role of preoperative CI in modulating the intraoperative EEG dynamics.

Our results are consistent with data linking CI to altered EEG patterns during anaesthesia. Gutiérrez et al. demonstrated that patients with lower MoCA scores exhibited reduced alpha-band activity during anaesthesia, which is indicative of deeper hypnotic states. [22] These findings are also in line with evidence that age-related brain changes, such as reduced cortical volume and diminished neurotransmitter synthesis, contribute to increased anaesthetic sensitivity in elderly patients [36, 37]. While this study did not perform spectral EEG analysis, the prolonged duration of NI < 40 observed in patients with severe CI may similarly reflect reduced alpha activity, as suggested by the Narcotrend algorithm. In contrast to our study design, anaesthesia was administered exclusively according to the age-adapted MAC and not with a target of NI. Furthermore, the EEG was recorded using a 16-channel EEG according to the international 10–20 system. In summary, anaesthetic doses did not differ between patients with and without CI. For the present dataset, no assessment of the alpha activity described by Gutiérrez et al. can be made with regard to cognition owing to the lack of EEG spectral analyses. In this respect, longer phases of too-deep anaesthesia with NI values < 40 were found in patients with severe cognitive deficit (MoCA score ≤ 21).

### Burst suppression

Interestingly, while BS was observed in 31.3% of the patients, no significant correlation was found between the occurrence of BS and CI. This is in contrast to studies suggesting that BS may reflect deeper stages of anaesthesia and increased vulnerability in cognitively impaired patients [13, 38]. This particularly affects elderly patients, who are more sensitive to anaesthetics and for whom evidence-based EEG monitoring is recommended by anaesthesiological societies, especially to prevent overdoses [3, 4].

Based on the above findings that prolonged periods of BS may adversely affect disease progression, and that a direct link between BS and cognitive deficits has not yet been described, Reese et al. postulated that BS is preceded by a state of reduced electrical activity (‘pre-burst suppression’). Even patients without BS can exhibit these phases of reduced electrical activity. Here, a higher percentage of ‘pre-burst suppression’ was associated with lower cognitive function. [39]

No correlation was found between the percentage of BS and cognitive deficits, but a correlation was found between the longer phases of low electrical activity (NI < 40). Nevertheless, our reliance on automated analysis should be considered a limitation. Based on the findings described above, however, it can be assumed that the results relating to BS in the present study are an underestimation of the actual time of BS owing to the lack of visual reanalyses of the raw EEG. Raw EEG traces were not manually reviewed, and our analyses therefore relied exclusively on the Narcotrend automated algorithm. This may have led to an underestimation of burst suppression events and represents a limitation of our study. In addition, recent research by Fleischmann et al. emphasised the limitations of automated BS detection algorithms and recommended visual EEG analysis for improved accuracy. [40] Further studies using Narcotrend monitoring and visual reanalyses are necessary to confirm this hypothesis and to investigate the relationship between BS and cognitive status. Future studies should also incorporate spectral EEG analyses, such as those proposed by Gutiérrez et al., to explore the contribution of alpha-band activity as an additional marker of cognitive vulnerability. [22]

### Secondary outcomes

Preoperative CI is associated with a higher risk of postoperative delirium, prolonged hospitalization, discharge to care facilities, falls, severe bleeding, and cardiopulmonary complications [1, 15, 41–45]. This study found that CI patients experienced significantly more pain requiring treatment (VAS), but there was no correlation with delirium, possibly due to the high CI prevalence (64.2%). Previous studies have reported lower CI rates of 24% and 21%, respectively [1, 41]. The incidence of delirium in this study (6%) was also lower than that reported in other studies (21–70%) [1, 33, 41, 42].

No study has directly examined the link between preoperative CI and postoperative pain, although older patients often receive inadequate pain management. CI complicates pain assessment and communication, likely leading to under-reported and under-treated pain [46]. The temporal pattern in our data, with significant differences observed at T1 and T3 but not at T2, may reflect multifactorial influences such as variable analgesic requirements, recovery dynamics, or circadian variation, and should be interpreted with caution. Surgical type and approach may also influence postoperative pain levels, as open procedures are generally associated with higher pain intensities compared to minimally invasive techniques. In our cohort, most patients underwent robot-assisted prostatectomy, which limited heterogeneity; however, surgical type cannot be ruled out as a potential contributor to variability in pain outcomes. Given the complex, multifactorial risks associated with CI, further research is needed to clarify their impact on postoperative outcomes [47].

### Limitations

This study had several limitations must be acknowledged. First, this single-centre study, conducted in a predominantly male cohort undergoing urological surgery, limits the generalisability of our findings to other populations and surgical specialties. As nearly all participants were older men, extrapolation to female patients or different surgical cohorts remains uncertain. Given reported sex-related differences in anaesthetic pharmacodynamics and EEG patterns, future multi-centre investigations including more diverse and balanced populations are warranted to confirm these observations. Although the observed associations reached statistical significance, their effect sizes were modest.

Consequently, the findings should be regarded as exploratory and hypothesis-generating, rather than definitive evidence of strong predictive value. Moreover, because analysing multiple time points inherently increases the probability of type I errors (i.e., false positives), all p-values were interpreted with particular caution. Second, the observational design precludes causal inferences regarding the relationship between CI and anaesthetic sensitivity. Third, while the Narcotrend monitor provides valuable insights into anaesthetic depth, its algorithm does not fully disclose the weighting of spectral components, such as alpha activity, which limits direct comparisons with studies using advanced EEG systems. The relationship between NI values (ranging from 100 for wakefulness to 0 for deep anaesthesia) and alpha activity is inconsistent as alpha activity assessment is more complex [48]. It has recently been shown that intraoperative EEG alpha power is independently associated with postoperative mortality and adverse outcomes [49]. While NI indices are used clinically to prevent over- or underdosing, their connection to reduced alpha activity remains unproven owing to the opacity of the algorithm, especially in elderly patients [39]. Further research on spectral analysis is needed to explain the difficulties in maintaining the target index values during anaesthesia. Importantly, we did not adjust for potentially relevant covariates such as pre-operative antihypertensive or psychotropic medications, intra-operative hypotension duration, EDA use, blood loss, perioperative temperature, or pre-existing cerebral small-vessel disease. This was primarily due to the moderate sample size, which limited the number of predictors that could be included without risking model overfitting, and the fact that some variables were not systematically collected in all patients. As a consequence, residual confounding cannot be excluded. Given the cross-sectional, observational study design, our findings demonstrate associations but cannot establish causality. Future prospective studies with larger sample sizes should incorporate relevant covariates into multivariable models, apply clearly defined patient groups, and include longitudinal assessment of intraoperative EEG parameters and postoperative outcomes to better control for confounding and enable more definitive conclusions on causal relationships. However, excluding patients with pre-existing neurological disease may have led to an underestimation of the prevalence of CI in this cohort. Finally, assessing pain in patients with cognitive impairment may be challenging, as self-reporting can be less reliable. The use of self-report pain scales in cognitively impaired patients introduces a risk of measurement bias, and it remains unclear to what extent the observed differences reflect true nociceptive vulnerability versus difficulties in pain assessment. Furthermore, although some intraoperative variables demonstrated statistically significant correlations with the occurrence of deep anaesthesia (NI < 40), the associated effect sizes were consistently small, indicating weak relationships. These findings emphasize the limited explanatory power of the examined variables within the current sample. Consequently, larger studies with increased sample sizes are warranted to validate these preliminary associations and to better elucidate potential contributing factors.

This study found prolonged deep anaesthesia (NI < 40) only in patients with severe cognitive impairment (MoCA score ≤ 21), whereas Gutiérrez et al. observed differences at an MoCA cut-off of 26 [22]. The high prevalence (64.2%) of cognitive deficits observed in this study may reflect patient anxiety before surgery. These findings, consistent with recent research, call into question the preoperative cut-off value of the MoCA test [25].

Questions about possible factors influencing EEG behaviour concern cerebral perfusion, which is normally subject to autoregulation, but also to disease-related changes [50]. Insufficient cerebral perfusion based on a low MAP causes a decrease in brain electrical activity with EEG slowing. This is also associated with a decrease in the index values of anaesthesia depth monitors [49].

Since, after excluding all patients with hypotension (MAP < 65 mmHg) in the overall time window, only a small number of 27 patients remained in the present follow-up, further studies with a larger number of cases and, at best, a study protocol are required to adequately assess this influencing factor.

Several studies have raised concerns about the reliability of automated systems for the detection of BS in EEG monitors. Fleischmann et al. recommended reviewing raw EEG in addition to the processed index values, as visual inspection of the raw data is considered more reliable for identifying BS episodes. [40] Their study of 90 patients (> 60 years) compared the detection of BS via a GE Entropy Module and a 10-channel EEG. While both methods identified suppression in 56 patients (62%), visual analysis detected 13 additional cases (14%) missed by the entropy module, likely owing to differences in suppression amplitude thresholds.

While the occurrence of BS was not significantly associated with MoCA scores in our cohort, this may, at least in part, reflect the known limitations of the Narcotrend algorithm in detecting BS, as automated indices can underestimate BS events compared with visual EEG analysis. Our reliance on automated analysis should therefore be considered a limitation, and future studies using manual EEG review and spectral analysis may provide more precise insights into the role of BS in patients with cognitive impairment. In general, this study highlights the need for EEG training and notes that current anaesthesia depth algorithms are not adjusted for age-related brain changes.

In addition, the follow-up period was limited, highlighting the need for further research.

Future research should focus on integrating multimodal monitoring approaches, including spectral EEG analysis and biomarkers of neuroinflammation, to better understand how CI influences anaesthetic sensitivity and postoperative outcomes. In clinical practice, also brief screening tools such as the Mini-Cog might represent a pragmatic alternative to the MoCA in busy preoperative settings. Additionally, randomised controlled trials assessing whether tailored anaesthetic protocols based on preoperative cognitive assessments can improve outcomes are required.

## Conclusion

Here, we demonstrated the importance of preoperative cognitive screening in elderly surgical patients. The association between severe CI and prolonged deep anaesthesia highlights the need for individualised anaesthetic management strategies to mitigate risks. Incorporating advanced EEG metrics into routine monitoring could enhance our ability to identify high-risk patients and optimise perioperative care.

## Supplementary Information

Supplementary Material 1: Supplement Table A

Supplementary Material 2: Supplement Table B

## Data Availability

The data that support the findings of this study are not openly available due to reasons of sensitivity and are available from the corresponding author upon reasonable request.
