# Supplementary material for: Preoperative cognitive impairment predicts deep anaesthesia and higher postoperative pain in elderly patients: an observational study
Source: BMC Anesthesiol. 2025 Oct 31;25:540. doi: 10.1186/s12871-025-03452-w (PMC12579417; doi:10.1186/s12871-025-03452-w)
Supplement: Supplementary file 1 — Supplementary Material 1: Supplement Table A [file 12871_2025_3452_MOESM1_ESM.docx]

| **Supplement Table A.** Correlations between MoCA test scores and demographic and intraoperative variables | | |
| --- | --- | --- |
| **Variable** | **ρ / η²** | **P** |
|  | *n*=67 |  |
| Age in years | -0.068 | 0.583 |
| Body mass index in kg/m^2^ | 0.062 | 0.618 |
| Surgical Apgar Score (SAS) | 0.249 | 0.051 |
| Epidural anaesthesia | 0.009 | 0.435 |
| Preoperative Hb in g dl^-1^ | 0.057 | 0.645 |
| Preoperative creatinine value in mg dl^-1^ | 0.025 | 0.843 |
| Duration of surgery (incision-suture) in minutes | 0.022 | 0.859 |
| Mean arterial pressure in mmHg | 0.040 | 0.747 |
| Intraoperative volume administered in ml | -0.120 | 0.333 |
| Blood loss in ml | -0.148 | 0.242 |
| Intraoperative minimal temperature in °C | 0.029 | 0.819 |
| Intraoperative maximal temperature in °C | 0.183 | 0.144 |
| Occurrence of intraoperative hypotension < 65 mmHg | 0.089 | 0.210 |
| ρ = Spearman's correlation coefficient; η² = eta-squared coefficient | |  |
