# Supplementary material for: Preoperative cognitive impairment predicts deep anaesthesia and higher postoperative pain in elderly patients: an observational study
Source: BMC Anesthesiol. 2025 Oct 31;25:540. doi: 10.1186/s12871-025-03452-w (PMC12579417; doi:10.1186/s12871-025-03452-w)
Supplement: Supplementary file 2 — Supplementary Material 2: Supplement Table B [file 12871_2025_3452_MOESM2_ESM.docx]

| **Supplement Table B.** Correlations between occurrences of NI <40 (yes/no) and demographic and intraoperative variables | | |
| --- | --- | --- |
| **Variable** | **η² / χ²** | **P** |
|  | *n*=67 |  |
| Age in years | 0.001 | 0.841 |
| Body mass index in kg/m^2^ | 0.021 | 0.248 |
| Surgical Apgar Score (SAS) | 0.034 | 0.153 |
| Epidural anaesthesia | 1,044 | 0.307 |
| Preoperative Hb in g dl^-1^ | 0.052 | 0.063 |
| Preoperative creatinine value in mg dl^-1^ | 0.003 | 0.642 |
| Duration of surgery (incision-suture) in minutes | 0.054 | 0.058 |
| Mean arterial pressure in mmHg | 0.02 | 0.252 |
| Intraoperative volume administered in ml | 0.069 | **0.032** |
| Blood loss in ml | 0.104 | **0.009** |
| Intraoperative minimal temperature in °C | 0.077 | **0.025** |
| Intraoperative maximal temperature in °C | 0.008 | **0.048** |
| Occurrence of intraoperative hypotension < 65 mmHg | 3.576 | 0.466 |
| η² = eta-squared coefficient; χ² = Chi-square test | |  |
